# Antiproliferative and Antiangiogenic Activities of Smenospongine, a Marine Sponge Sesquiterpene Aminoquinone

**DOI:** 10.3390/md9020154

**Published:** 2011-01-28

**Authors:** Dexin Kong, Takao Yamori, Motomasa Kobayashi, Hongquan Duan

**Affiliations:** 1 School of Pharmaceutical Sciences and Research Center of Basic Medical Sciences, Tianjin Medical University, Tianjin 300070, China; Email: duanhq@tijmu.edu.cn; 2 Division of Molecular Pharmacology, Cancer Chemotherapy Center, Japanese Foundation for Cancer Research, 3-10-6, Ariake, Koto-ku, Tokyo 135-8550, Japan; Email: yamori@jfcr.or.jp; 3 Graduate School of Pharmaceutical Sciences, Osaka University, Yamada-oka 1-6, Suita, Osaka 565-0871, Japan; Email: kobayashi@phs.osaka-u.ac.jp

**Keywords:** smenospongine, antiangiogenesis, antiproliferation

## Abstract

We previously reported that smenospongine, a sesquiterpene aminoquinone isolated from the marine sponge *Dactylospongia elegans*, showed antiproliferative or cytotoxic activities on leukemia cells. In this study, we investigated the effect of smenospongine on solid tumors. Since angiogenesis is well known to be closely involved in growth and metastasis of solid tumors, the antiangiogenic effect of smenospongine was determined. We found that smenospongine inhibited proliferation, migration and tube formation of human umbilical vein endothelial cells (HUVEC). Moreover, the inhibitory activity of smenospongine on growth of solid tumor cells was investigated. Smenospongine inhibited the growth of 39 human solid cancer cells *in vitro*, with a mean Log GI_50_ value of −5.55. In conclusion, smenospongine exhibits antitumor activity on solid tumors via two mechanisms, an antiangiogenic effect on endothelial cells and direct inhibition of growth of tumor cells.

## 1. Introduction

Angiogenesis, the process of generating new blood vessels from a primitive vascular network, is known to be required for the growth and metastasis of solid tumors. After the size reaches 1 mm^3^, a tumor cannot continue to grow without angiogenesis [1]. Therefore, antiangiogenesis has become known as an effective approach for therapy of solid tumors. Correspondingly, since the FDA (U.S.) approval of avastin in 2004 (a recombinant human monoclonal antibody against VEGF), more than 30 inhibitors of angiogenesis have been either approved or are in clinical trials for cancer therapy [1,2,3].

**Figure 1:**
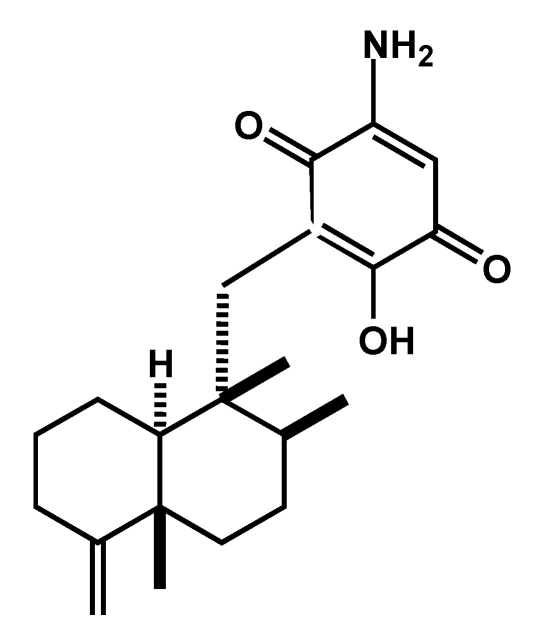
Chemical structure of smenospongine.

We previously reported that smenospongine (Figure 1), a sesquiterpene aminoquinone isolated from the Indonesian marine sponge *Dactylospongia elegans*, showed multifaceted antitumor activities on leukemia cells [4,5,6]. Thus, smenospongine induced G1 arrest in chronic myelogenous leukemia (CML) cells and apoptosis in acute myelogenous leukemia (AML) and lymphocytic leukemia [4,6]. Here, we present our investigations of the effect of smenospongine on solid tumors with a focus on the antiangiogenic effect. In addition, the inhibitory effect on the growth of various cancer cells is examined. 

## 2. Results and Discussion

### 2.1. Smenospongine Inhibits Proliferation of Human Umbilical Vein Endothelial Cells (HUVECs)

Angiogenesis is a multi-step process involving basement membrane dissolution, endothelial cell proliferation and migration, and new basement membrane formation [7]. To examine the antiangiogenic activity of smenospongine, we first determined the activity on the proliferation of HUVECs by use of WST-8 assay as reported by us previously [3]. 

**Figure 2:**
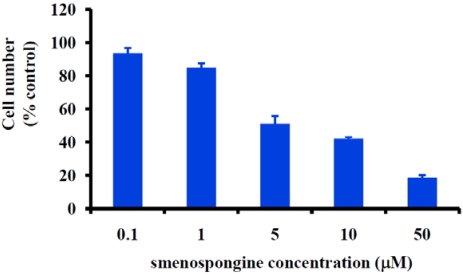
Effect of smenospongine on the proliferation of human umbilical vein endothelial cells (HUVEC).

As shown in Figure 2, smenospongine inhibited the proliferation of HUVECs in a concentration-dependent manner. The IC_50_ value was calculated to be 4.9 μM (95% confidence interval: 1.5 to 16 μM) by use of GraphPad Prism 4. 

### 2.2. Smenospongine Blocks HUVEC Migration

**Figure 3:**
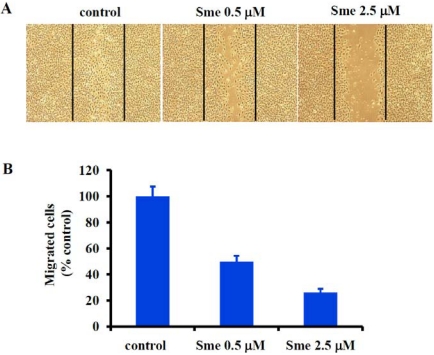
Effect of smenospongine on HUVEC migration. (**A**) Representative wound healing graphs of HUVECs treated with DMSO (control) or various concentrations of smenospongine (Sme); (**B**) Percentage of HUVECs migrated following treatment with various concentrations of smenospongine (Sme) relative to DMSO treatment alone.

To further investigate the antiangiogenic activity of smenospongine, the effect on migration of HUVECs was examined by use of a wound healing assay as reported [8,9]. Figure 3 shows the representative wound healing graphs of HUVECs treated with DMSO (control) or various concentrations of smenospongine for 18 h. As indicated, smenospongine blocked the migration of HUVECs in a concentration-dependent manner, suggesting potential antiangiogenic activity.

### 2.3. Smenospongine Inhibits Capillary-like Tube Formation by HUVECs

**Figure 4:**
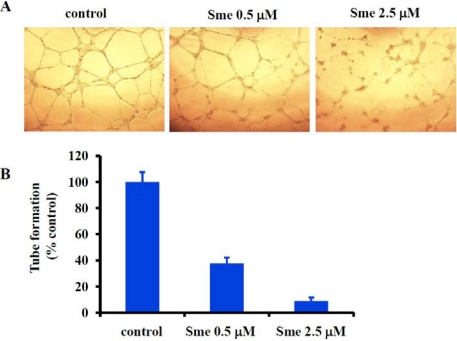
Effect of smenospongine on tube formation by HUVECs. (**A**) Representative images depicting the tube formation by HUVECs treated with DMSO (control) or various concentrations of smenospongine (Sme); (**B**) Percentage of tube formation by HUVECS following treatment with various concentrations of smenospongine (Sme) relative to DMSO treatment alone.

Finally, we examined the effect of smenospongine on the *in vitro* tube formation by HUVECs as reported by us previously [3]. The HUVECs were incubated in the absence or presence of 0.5 or 2.5 μM smenospongine. Eighteen hours later, the capillary-like tube structure formation on matrigel was observed under a microscope. The representative networks of tube structures are shown in Figure 4. Smenospongine inhibited tube formation in a concentration-dependent manner, further suggesting the promising antiangiogenic activity.

### 2.4. Smenospongine Inhibits Growth of Various Solid Tumor Cells

**Figure 5:**
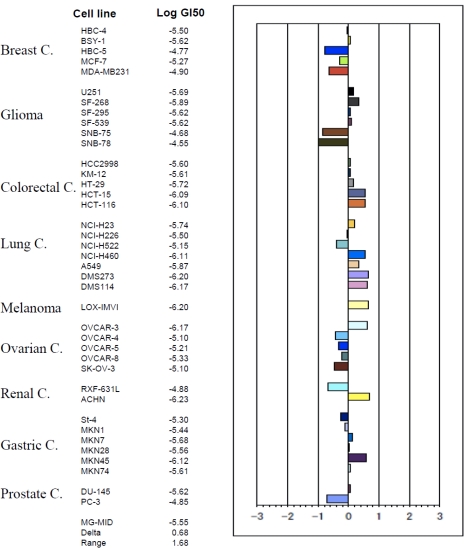
Effect of smenospongine on cell growth of 39 tumor cell lines. The Log GI_50_ values of smenospongine for the cell lines in JFCR39 panel, and the JFCR39 fingerprint which is plotted based on the Log GI_50_ values [10], are indicated. In the JFCR39 fingerprint, the X-axis shows difference in logarithmic scale between the mean of Log GI_50_ values for all 39 cell lines (MG-MID, expressed as 0 in the fingerprint) and the Log GI_50_ for each cell line in JFCR39 panel. Columns to the right of 0 indicate the sensitivity of the cell lines to smenospongine and columns to the left indicate the resistance. MG-MID: mean of Log GI_50_ values for all 39 cell lines; Delta: difference between the MG-MID and the Log GI_50_ value for the most sensitive cell line; Range: difference between the Log GI_50_ values for the most resistant cell line and the most sensitive cell line.

To investigate the direct activity on solid tumor cells, we determined the inhibitory effect on the cell growth of 39 solid tumor cell lines (JFCR39) by use of the sulforhodamine B (SRB) assay, as described by us previously [10]. The GI_50_ values (the concentrations of given compounds to cause 50% growth inhibition of cells) for each tumor cell line were obtained and the JFCR39 fingerprint was plotted based on the Log GI_50_ values [8,10,11] (Figure 5). The mean Log GI_50_ for all the 39 tumor cell lines was calculated to be −5.55. Among the cell lines, those of colorectal cancers and most lung cancers showed relatively higher sensitivity while those of most ovarian and breast cancers indicated relative resistance, suggesting such type of compound might be more applicable for treatment of the colorectal and lung cancers, than the ovarian and breast cancers, when approved as an anticancer drug in the future.

## 3. Experimental Section

### 3.1. Isolation and Identification of Smenospongine

Smenospongine was isolated from the marine sponge *Dactylospongia elegans* (collected in Indonesia in 2001) as described previously [4]. The structure of smenospongine was identified by comparison of the mass and NMR data with those reported [12]. 

### 3.2. Cell Lines and Cell Culture

HUVECs were purchased from Clonetics and maintained in endothelial cell growth medium-2 (EGM2 BulletKit, Conetics) at 37 °C in a humidified atmosphere containing 5% CO_2_.

A panel of 39 human cancer cell lines termed JFCR39 [8,10], which consists of the following cell lines: lung cancer, NCI-H23, NCI-H226, NCI-H522, NCI-H460, A549, DMS273 and DMS114; colorectal cancer, HCC-2998, KM-12, HT-29, HCT-15 and HCT-116; gastric cancer, MKN-1, MKN-7, MKN-28, MKN-45, MKN-74 and St-4; ovarian cancer, OVCAR-3, OVCAR-4, OVCAR-5, OVCAR-8 and SK-OV-3; breast cancer, BSY-1, HBC-4, HBC-5, MDA-MB-231 and MCF-7; renal cancer, RXF-631L and ACHN; melanoma, LOX-IMVI; glioma, U251, SF-295, SF-539, SF-268, SNB-75 and SNB-78; prostate cancer, DU-145 and PC-3, were cultured in RPMI 1640 medium supplemented with 5% fetal bovine serum and kanamycin (100 U/mL) at 37 °C in a humidified atmosphere containing 5% CO_2_.

### 3.3. WST-8 Assay

Cell viability was determined using the WST-8 assay kit (Kishida Chemicals, Japan) as described previously by us [3]. To investigate the effect of smenospongine on the growth of HUVECs, 0.1 mL of cells (5 × 10^3^ cells/well) was seeded in 96-well plate and incubated in EGM2 medium at 37 °C in a humidified atmosphere containing 5% CO_2_. Twenty-four hours later, 0.5 μL of various stock solutions of smenospongine or DMSO was added. After further incubation for 72 h, 10 μL of WST-8 was added to each well and further incubated. Three hours later, the absorbance at 450 nm was measured. The number of viable cells after treatment was calculated using the following formula: Cell number (% control) = 100 × (absorbance of a given sample − absorbance of Blank well)/(absorbance of Control well − absorbance of Blank well), where the Blank well contained medium but no cells and the Control well contained cells but no smenospongine. The IC_50_ value was calculated by fitting the data points to a logistic curve using the GraphPad Prism 4 software. Three independent experiments were carried out.

### 3.4. Wound Healing Assay for Cell Migration

HUVECs were allowed to grow to full confluence in 6-well plates at 37 °C in a humidified atmosphere containing 5% CO_2_. Then, the monolayer cells were scratched with a 1 mL pipette tip. Fresh EGM2 media were added with DMSO or with various concentrations (0.5, 2.5 μM) of smenospongine. After further incubation for 18 h, the migration was observed under microscope and photographs were taken. For quantification, the migrated cells were counted manually, and the percentage inhibition was expressed by comparison with the control (treated with DMSO). Two independent experiments were carried out.

### 3.5. *In Vitro* Assay for Capillary-like Tube Formation

The effect of smenospongine on tube formation *in vitro* was determined following the method reported by us previously [3]. Briefly, matrigel (BD Biosciences, CA) was thawed overnight at 4 °C. For coating, 50 μL of matrigel was pipetted into each well of pre-chilled 96-well plate, and then the plate was incubated at 37 °C for 45 min. 0.1 mL of HUVECs (1 × 10^5^ cells/mL) was seeded in each well of the coated plate. 0.5 μL of various stock solutions of smenospongine was added to achieve desired final concentrations and the cells were then incubated for 18 h at 37 °C. The capillary-like tubes formed were visualized under microscope. Representative network of tube structures formed in each well was photographed. The length of the tubes was measured for quantification, and the percentage of tubes formed in each smenospongine-treated well relative to the control well (DMSO-treated cells) was calculated. Representative data from 2 independent experiments, each performed in triplicate, were used for analysis.

### 3.6. Determination of Inhibitory Activity on Cell Growth of 39 Cancer Cell Lines and Plotting of JFCR39 Fingerprint

Inhibition of cell growth of cancer cells was assessed by the change in total cellular protein following 48 h of treatment with smenospongine, and was measured by SRB assay as described previously [8,10,11]. The concentration of smenospongine required for 50% growth inhibition (GI_50_) of cells was calculated. The graphic representation (termed fingerprint), the mean differential growth inhibition for the cells used in the JFCR39 panel, was plotted based on a calculation that uses a set of Log GI_50_ values [8,10,11]. 

## 4. Conclusions

Smenospongine inhibited proliferation, migration and tube formation of human endothelial cells, suggesting favorable antiangiogenic activity. Moreover, this compound inhibited growth of 39 cell lines of human solid tumors. Such results suggest smenospongine exhibits potential antitumor efficacy on solid tumors. To our knowledge, this is the first report about the antiangiogenic activity of sesquiterpene quinines, while some cytotoxic activities of such compounds were reported [13]. Smenospongine might be used as a lead compound for finding a promising anticancer drug candidate after structure-modification.
